# Proof-of-concept study of electrospun PLGA membrane in the treatment of limbal stem cell deficiency

**DOI:** 10.1136/bmjophth-2021-000762

**Published:** 2021-07-26

**Authors:** Charanya Ramachandran, Pallavi Deshpande, Ilida Ortega, Farshid Sefat, Rob McKean, Mala Srivastava, Sheila MacNeil, Sayan Basu, Virender Singh Sangwan

**Affiliations:** 1 Centre for Ocular Regeneration, Brien Holden Eye Research Centre, LV Prasad Eye Institute, Hyderabad, India; 2 Insitute of Population Healthy, University of Liverpool, Liverpool, UK; 3 School of Clinical Dentistry, The University of Sheffield, Sheffield, UK; 4 Biomedical and Electronics Engineering Department, University of Bradford, Bradford, UK; 5 The Electrospinning Company Ltd, Didcot, UK; 6 Nextvel Consulting LLP, Bengaluru, India; 7 Department of Materials Science and Engineering, The University of Sheffield, Sheffield, UK; 8 Cornea and Innovation, Dr Shroff's Charity Eye Hospital Delhi, New Delhi, India

**Keywords:** clinical trial, cornea, ocular surface, stem cells

## Abstract

**Objective:**

The aim of this study was to assess the safety of poly-lactic co-glycolic acid (PLGA) electrospun membranes as carriers for limbal tissue explants for treatment of limbal stem cell deficiency (LSCD).

**Methods and analysis:**

Approval was obtained for a first in-man study from the Drug Controller General of India. PLGA membranes were applied to the affected eye of five patients after removal of the vascular pannus. Simple limbal epithelial transplantation was performed and limbal explants were secured on the membrane using fibrin glue followed by a bandage contact lens. Patients were followed up for 1 year with ocular exams including slit lamp exam, corneal thickness measurements, intraocular pressure measurements and recording of corneal vascularisation and visual acuity. Systemic examinations included pain grading, clinical laboratory assessment, blood chemistry and urine analysis at baseline, 3 and 6 months after surgery.

**Results:**

PLGA membranes completely degraded by 8 weeks post-transplantation without any infection or inflammation. In all five patients, the epithelium regenerated by 3 months. In two in five patients, there was a sustained two-line improvement in vision. In one in five patients, the vision improvement was limited due to an underlying stromal scarring. There was recurrence of pannus and LSCD in two in five patients 6 months after surgery which was not attributable to the membrane. The ocular surface remained clear with no epithelial defects in three in five subjects at 12 months.

**Conclusion:**

PLGA electrospun membranes show promise as carrier for limbal epithelial cells in the treatment of LSCD.

**Key messages**

**What is already known about this subject?**

- Poly-lactic co-glycolic acid (PLGA) has long been used as suture material in surgeries and is known to be safe and biologically compatible. PLGA breaks down by hydrolysis into its basic components that we know do not elicit any local or systemic adverse reactions. We have shown in our previous publications that the PLGA supports limbal stem cell growth and transfer to the ocular surface in a predictable manner. Toxicity studies in rabbits indicated that the material was not toxic to the corneal epithelial cells and did not elicit any ocular or systemic adverse reactions.

**What are the new findings?**

- We are reporting the use of electrospun PLGA membranes as carrier for the transplantation of limbal epithelial cells. Here we show for the first time the usage of PLGA as a carrier for transplanting limbal cells to the ocular surface of human subjects for the treatment of limbal stem cell deficiency. In all patients, an intact ocular surface was regenerated by 3 months and vision improved in 60% patients. There were no safety concerns and membrane degradation was predictable.

**How might these results change the focus of research or clinical practice?**

- The material has the potential to replace the human amniotic membrane in the future thus making the technique of corneal surface regeneration accessible to more surgeons and hence more patients.

## Introduction

The corneal epithelium is a constantly renewing surface with a reserve of stem cells located at the limbus that allows for epithelial homeostasis to be maintained.^1 2^ The limbal region also separates the avascular transparent cornea from the adjoining vascular conjunctiva. When there is a loss of stem cells (eg, injury), the corneal epithelium is replaced by the conjunctival cells which form a cloudy, vascularised membrane over the eye leading to painful vision loss.^3^ This condition is referred to as limbal stem cell deficiency (LSCD) which can be classified as partial or total and unilateral or bilateral.

Total LSCD is treated by surgical removal of the vascularised pannus to be replaced with healthy epithelial cells. There are two effective methodologies to restore the stem cell population—the use of laboratory expanded cultured cells (cultured limbal epithelial cells, CLET)^4^ or simple limbal epithelial transplantation (SLET).^5^ SLET uses a biopsy of healthy limbal tissue which is cut into small pieces and held on human amniotic membrane (hAM) using fibrin glue. The cells from the limbal explants grow out and regenerate the epithelium without requiring laboratory expansion.^5^

SLET has greatly reduced the cost and resources required for regenerating a healthy corneal surface making it more accessible to many surgeons and patients. A recent health economics study showed the cost of SLET in India to be approximately 10% of the cost of CLET (Thokala *et al*, Economic, clinical and social impact of SLET for LSCD, In Press). The reported survival rate after CLET is 70% which is comparable to the SLET procedure.^4 6^ SLET allows for repeat surgeries to be performed without affecting the healthy donor eye^5^ and has been found to be more effective in treating children.^7^ Thus, SLET is fast becoming an accepted method of treatment for LSCD.^8^

The substrate used for transferring the cells to the denuded corneal surface is an important determinant of treatment outcome. Several cell carrier materials have been explored without significant side effects including fibrin glue,^9^ collagen,^10 11^ synthetic polymer membrane^12^ and hAM.^13 14^ Of these, hAM remains the material of choice for treating LSCD. hAM serves as a good material due to its anti-inflammatory properties, however, it requires sourcing, preparing, and storing under accredited tissue bank conditions. Even with good banking practice, hAM carries some risk of transferring virus or other pathogens to recipients. There is inherent variation in the quality and preparation of hAM and not every centre has access to a tissue bank thus greatly limiting its availability.

We developed a synthetic material with a long shelf-life, easy to manufacture, sterilise and scale up and breaks down in a safe and predictable manner.^15^ The membranes used in the clinical study were made of a copolymer (50:50) of poly-lactic co-glycolic acid (PLGA) based on an electrospinning protocol published earlier.^16^ PLGA was chosen because of its biocompatibility and because it has been extensively used as absorbable sutures for ophthalmic surgery.^17^ In vitro studies provided evidence of the PLGA membrane’s ability to support the expansion of the limbal stem cell population in culture. Using ex vivo rabbit cornea models we showed that the membranes allowed successful transfer of these cells to a damaged cornea.^15^ Toxicology studies in rabbits confirmed the absence of local or systemic toxic effects and showed that the membranes disappeared completely within 28 days.^18^

After these satisfactory preclinical studies, our next step was to conduct a proof-of-concept trial in man. The study objectives were to assess the performance of the PLGA membranes in terms of their safety (primary outcome) and efficacy (secondary outcome) in restoring the health of the ocular surface and vision in patients with LSCD.

## Materials and methods

### Study methodology

This was an open-label, non-randomised, single-arm, single centre (LV Prasad Eye Institute (LVPEI)) proof-of-concept validation study. The trial was registered with the Clinical Trials Registry India (CTRI)-Registration Number CTRI/2015/08/006147 and clinicaltrials.gov (ClinicalTrials.gov Identifier: NCT02568527). Informed consent was obtained after the procedures involved in the study were explained to the patient by the investigator. The patient was then allowed time to consider the information before signing the consent form to indicate that they fully understood the information, and willingly volunteered to participate in the study. Translated versions of the informed consent document were used where required. Patients and public were not involved in the design, conduct, or reporting, of our research as it was not appropriate for the study.

### Selection criteria

#### Inclusion criteria

Participants ≥18 years of age, clinically confirmed diagnosis of unilateral total LSCD due to chemical injury, no prior history of limbal transplantation, absence of other active ocular pathologies and ability to provide written informed consent.

#### Exclusion criteria

Bilateral or partial LSCD, LSCD due to autoimmune disorders, previous neoplastic/cancer disease, severe dry eyes, acute systemic infections, prior history of limbal transplantation or multiple surgeries in the limbal region, uncontrolled diabetes, pregnant and lactating women and participation in any other investigational trial within 30 days prior to screening for this study.

The planned sample size was 10 subjects, but only five were enrolled as the entry criteria were stringent and there was a defined period in which to store and use these membranes for this study. An amendment was submitted to and approved by the DCGI which allowed us to relax the inclusion criteria to include patients with partial LSCD. However, by the time five patients were enrolled, it was close to the use by date for the membranes beyond which period no new patients could be enrolled. The decision was taken to run the study with five patients reporting on each one fully.

### Membrane production, sterilisation and shipping

Membranes were produced by The Electrospinning Company, UK using PLGA (Purac, The Netherlands) of molecular weight 44 kg/mol with a 50:50 ratio of lactide to glycolide. The membranes were 50 µm thick with fibre diameters of 2–3 µm.^15^ Each membrane was placed in a small container and vacuum sealed in a medical grade bag along with desiccant and a humidity indicator strip. Membranes were then sterilised via gamma-irradiation at Synergy Health Plc. (Moray Road, Swindon, UK), with an external dose range of 25–40 kGy.^19^ These were then shipped on dry ice via World Courier services to LVPEI. Once received, membranes were stored at −20°C until surgery.

**Figure 1:**
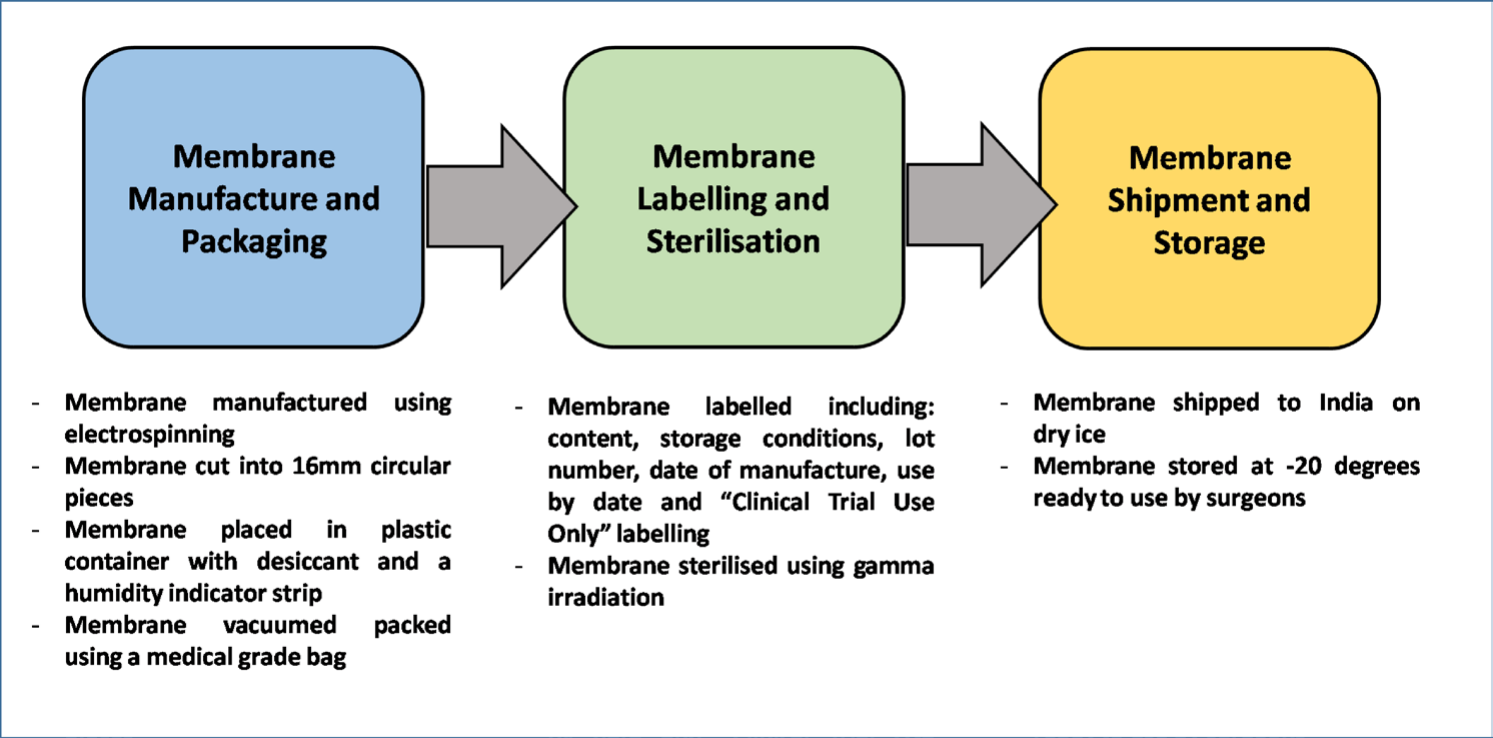
Schematic detailing the steps followed to deliver the PLGA electrospun membranes to surgeons in India including packaging, sterilisation, shipping, and storage procedures. PLGA, poly-lactic co-glycolic acid.

The humidity within the package was noted prior to opening the seal to ensure the integrity of the PLGA membranes as our previous study showed that humidity readings of >50% indicated PLGA degradation. The membranes were to be used in patients only if the humidity was <30% (figure 1).

### Screening and surgical procedure

Patients were screened prior to surgery and were assigned a unique identification number. Screening included assessment of both ocular and systemic parameters. The fibrovascular pannus covering the cornea was removed with sharp and blunt dissection, and any bleeding vessels were cauterised with bipolar cautery. A sterile PLGA membrane was then secured in place on the denuded cornea using fibrin glue (Tisseel, Baxter India, India). A 2×2 mm strip of limbal tissue was taken from the healthy eye and divided into 8–10 pieces and distributed evenly over the PLGA membrane on the eye. Finally, a soft bandage contact lens (Johnson & Johnson Vision Care, Florida, USA) was placed on the eye. Patients were monitored on day 1 postsurgery in the hospital and then discharged. Patients were monitored for any adverse events at every follow-up visit using the procedures as listed in online supplemental table 1.

10.1136/bmjophth-2021-000762.supp1 Supplementary data

## Results

Patient’s demographics are listed in table 1.

**Table 1:** Patient demographics

Patient ID	Sex	LSCD type	Duration of injury	Cause of LSCD	Prior interventions for LSCD	Systemic diseases
MTR01	Male	Total	6 months	Acid burn	None	None reported
MVR02	Male	Total	4 years	Chemical injury	None	None reported
AM03	Female	Total	24 years	Lime injury	None	Gastritis, anaemia
KM04	Male	Total	6 months	Chemical injury	None	Diabetes
BS05	Male	Total	10 years	Idiopathic onset	None	Diabetes, hypertension

LSCD, Limbal Stem Cell Deficiency.

### Subject 1 (MTR01)

A male patient presented to the clinic with an acid injury in the left eye resulting in total LSCD. His vision at presentation was hand movements with perception of light. The patient was on anti-inflammatory and anti-glaucoma medication in the left eye. The intraocular pressure (IOP) in the left eye was noted to be high (digitally). Lens echo and an acoustically clear vitreous could be seen in the B-scan with normal axial length and attached retina. Optical coherence tomography was used to measure the thickness of the cornea and it was 490 µm. The patient was posted for transplantation surgery with conjunctival autograft, for treating the symblepharon.

At 1-week follow-up, the PLGA membrane, limbal biopsies and superior conjunctival autograft were noted. At 1 month, the IOP continued to be high (digital assessment), so the patient was started on anti-glaucoma medications (online supplemental table 4). Most of the PLGA membrane had dissolved with only residual fragments evident. Fluorescein staining showed that the ocular surface was smooth with no noticeable epithelial defects. Two months after surgery, the patient presented with conjunctival congestion and vision was perception of light. The PLGA membrane had completely dissolved by this time. Fluorescein staining revealed microcystic oedema and superficial punctuate keratitis (SPKs). The IOP was high at 30 mm Hg and a dense total cataract was recorded. The patient was continued on antiglaucoma medications.

**Figure 2:**
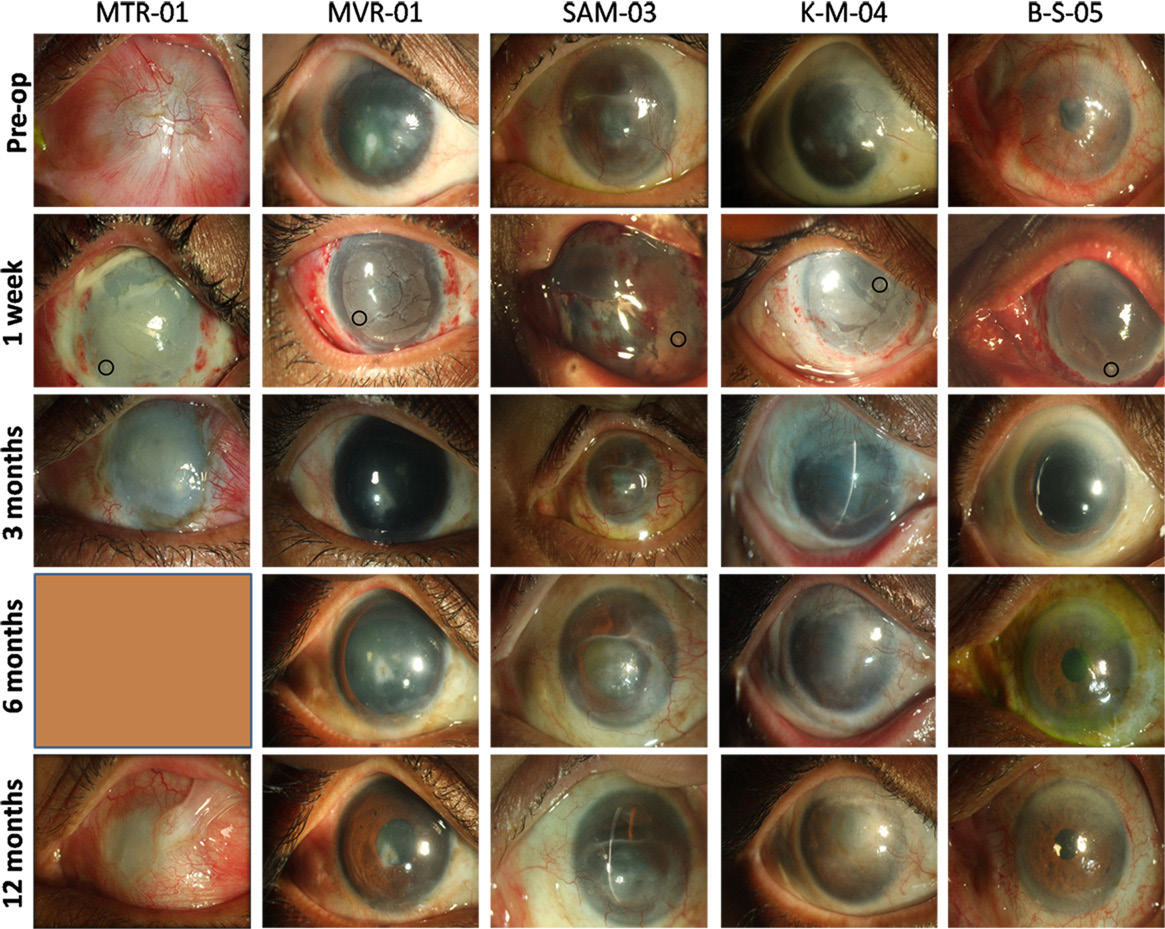
Shown in the figure are images of the treated eye pre-treatment and post-treatment for the five patients. The circles at 1 week indicate the location of the explants on the ocular surface. The PLGA membrane could not be detected in the 3month visit indicating its complete breakdown. PLGA, poly-lactic co-glycolic acid.

At 3 months, the IOP was 32 mm Hg and fluorescein staining showed presence of SPKs and microcystic oedema. Tear secretion, vitals and clinical laboratory assessments were normal. At the final follow-up, recurrence of LSCD was confirmed with 360° vascularisation, partial conjunctivalisation and superior symblepharon (figure 2). IOP in the left eye was approximately 28 mm Hg. B-scan revealed disc evacuation (online supplemental figure 2). The vitals and clinical laboratory assessments were normal (online supplemental table 5).

There were no adverse events noted in this patient attributable to the PLGA membrane. The recurrent LSCD was likely due to increased IOP which did not allow the regenerated epithelium to survive. The donor eye was normal with no signs of deficiency.

### Subject 2 (MVR02)

A male patient presented a history of chemical burns in the right eye. The vision was counting fingers at 2 m. Lid drooping was noted and slit lamp examination showed conjunctival congestion with inferior epithelial defect and scar. The IOP was digitally normal, however, the patient had a thin cornea (493 µm). B-scan showed that the vitreous was clear and the retina was attached.

At 1 week, lid oedema and conjunctival congestion were noted. Corneal thickness in the right eye was reduced to 462 µm. At the 1month follow-up, the patient’s vision was 20/80 and improved to 20/50 with pin hole. Mild conjunctival congestion was noted and IOP was 14 mm Hg. Scarring in the central cornea became apparent. At 2 months, the patient had a visual acuity of 20/60 which improved to 20/50 with correction (online supplemental table 2). The PLGA membrane had completely dissolved and the fluorescein staining showed a stable and healthy epithelium. Tear secretion was normal (online supplemental table 3). IOP was 19 mm Hg in the right eye and the fundus was normal. At 1 year, the ocular surface had regenerated well (figure 2), vision improved to 20/40 with correction, and the fundus was normal. Vitals and clinical chemistry values were normal (online supplemental table 5).

There were no adverse events noted. The vision improved by >2 lines but did not reach 20/20 because of the central scar. The donor eye was normal with no signs of deficiency.

### Subject 3 (AM03)

A female patient presented with reduced vision following injury when she was 2 years old. Vision in the affected eye was counting fingers at 1 m. The cornea was conjunctivalised and vascularised with a superior scar and few SPKs centrally with a region of clear inferior cornea. IOP was assessed to be normal digitally and B-scan was normal. The corneal thickness in the right eye was 630 µm.

At 1 week, the membrane and limbal explants were intact. At 1 month, mild conjunctival congestion was noted in the right eye and most of the PLGA membrane had degraded. Fluorescein staining showed a clear corneal surface without any defects. Slit lamp examination confirmed the presence of a superior scar with residual clear cornea. The corneal thickness was 440 µm. The IOP was 18 mmHg and B-scan revealed a well attached retina. At 2 months, the vision had improved to counting fingers at 2 m. Fluorescein staining revealed the presence of a large epithelial defect (>8 mm). Central tarsorrhaphy was performed and a bandage contact lens was applied to aid with the healing of the defect.

The patient presented at 6 months’ post-surgery with pain, watering, and photophobia in the right eye. The tarsorrhaphy was removed and vision was recorded to be counting fingers at 2 m. The cornea had a superior scar with vascularisation noted as recurrent LSCD (figure 2). B-scan (online supplemental figure 2), vitals and clinical chemistry values were normal at this visit (online supplemental table 5). At the final visit, the patient did not have pain or photophobia. The vision in the right eye had improved to 20/400.

There were no adverse events noted. There was partial recurrence of LSCD. The vision improved substantially from counting fingers to 20/400 but was limited because of the stromal scar and recurrent LSCD. The donor eye was normal with no signs of deficiency.

### Subject 4 (KM04)

A male patient presented with decreased vision in the left eye. Slit lamp examination showed extensive scarring, vascularisation and thinning of the cornea (310 µm) in the left eye. B-scan was normal.

At 1 month after surgery, the vision was counting fingers at 20 cm. The ocular surface was irregular, but no epithelial defect was noticed. The corneal thickness was 260 µm and IOP was 18 mmHg. At 2 months, the vision had improved to counting fingers at 1 m (online supplemental table 2). Microcystic oedema and extensive central scar were noted. The lens was cataractous, IOP was 19 mm Hg and B-scan was normal. At 3 months, the patient presented with occasional mild pain in the left eye. The cornea was irregular with microcystic oedema. However, there were regions of clear cornea compared with pre-surgery. The vision remained unaltered with normal IOP, tear secretion and retinal status. The visual and ocular status of the patient remained unaltered in the subsequent 6 months and 1year follow-up except for the reduction of microcystic oedema (figure 2). Mild non-proliferative diabetic retinopathy was clinically evident at 6 months probably due to the clearing of the cornea. This was attributed to the diabetes and hypertension which the patient presented with before the surgery.

There were no other adverse events noted due to the PLGA. The vision had improved but was limited by the extensive corneal scar and cataract. The donor eye was normal with no signs of deficiency.

### Subject 5 (BS05)

A male patient presented with cloudy vision in the left eye with intermittent pain and watering. There was no prior history of inflammation, autoimmune disorder, chemical injury, surgery or infection that led to the onset of LSCD in this patient. Having confirmed that the onset was idiopathic, which is unlikely to alter the outcomes, we included this subject in our trial though this was not explicitly stated in the inclusion criteria. Since the vision in the affected eye was 20/400. Fluorescein staining showed the presence of a few SPKs in the right eye and 360° conjunctivalisation and vascularisation in the left eye. The IOP and B-scan were normal. The corneal thickness was 500 µm in the left eye.

At 1 week, the PLGA membrane was intact and the IOP was assessed to be normal. At 1 month, the patient presented with a vision of 20/200. Fluorescein staining showed a large epithelial defect of 8 mm. Tarsorrhaphy was done to aid healing of epithelium. At 3 months, the patient was asymptomatic and the tarsorrhaphy was left intact. Slit lamp examination showed a clear and well regenerated ocular surface with no residual epithelial defect at 6 months (figure 2). The vision had improved to 20/60 (online supplemental table 2). At the final follow-up, the patient presented with vision of 20/80. All other ocular parameters (online supplemental figures 1 and 2) and vitals and clinical chemistry values were normal (online supplemental table 5).

There were no adverse ocular or systemic findings in this patient. The vision in this patient improved by >2 lines. The donor eye was normal with no signs of deficiency.

The findings in each patient is summarised in table 2.

**Table 2:** Summary of findings

Patient ID	BCVA		Corneal thickness (microns)		Schirmer’s Test (mm)		IOP (mm Hg)		Fundus	Findings Post-Sx	
	Pre-Sx	Post sx	Pre-Sx	Post sx	Pre-Sx	Post sx	Pre-Sx	Post sx		Recipient eye	Donor eye
MTR01	HM	PL	830	900	27	19	Dig. High	28	Disc excavation	Advanced cataract glaucomatous disc damage	Normal
MVR02	CF-2m	20/40	527	462	18	15	Dig. normal	14	Normal	Stromal scarring	Normal
AM03	CF-1m	20/400	630	538	20	24	Dig. normal	15	Normal	Superior scar with recurrent partial LSCD	Normal
KM04	HM	CF-2m	310	260	18	24	15	18	Normal	Stromal scarring and cataract	Normal
BS05	20/400	20/80	564	445	20	14	19	10	Normal	Stromal scarring	Normal

BCVA, Best Corrected Visual Acuity; IOP, Intraocular Pressure; LSCD, Limbal Stem Cell Deficiency.

### Membrane handling and breakdown

The breakdown of the membrane was predictable and complete by 8 weeks. The surgeon noted that the membranes were more brittle to handle than hAM. While this did not affect the ability of the membranes to support cell outgrowth from limbal explants this was noted by the surgeons as an area for improvement (discussed below).

## Discussion

The purpose of the proof-of-concept study was to obtain confirmation of the safety of a PLGA membrane to be used in treating LSCD. These studies are usually based on a very small number of patients but have particular value because of the opportunity they present to learn about the new biomaterial or technology at an early stage and to improve on it if needed.

In this five-patient study, we demonstrated that there were no adverse effects of using the PLGA membrane on the cornea. As expected, we found that the membrane had lost most of its mass by 4 weeks and had completely disappeared by 8 weeks; this corroborates our previous findings.^15^

The membrane did support the regeneration of a new corneal epithelium from limbal explants which was evident in all patients but was only sustained in three out of the five patients. Several of these patients presented with stromal scars which became evident after surgery. It was therefore not surprising that improvement in vision was limited.

When it came to selecting patients for the study, there were strict entry requirements to assess the performance of the membrane. A problem with recruiting for the study was that patients eligible for SLET using hAM were being treated rapidly in this hospital at this time essentially reducing the pool of patients available for this safety study.

Though this study was not designed to directly compare the performance of the PLGA membrane to the more commonly used hAM in LSC transplantation, this study has demonstrated that the PLGA membrane provides sufficient support for the limbal cells to form a stable ocular surface after transplantation. While there are several other materials that have shown promise as carriers for limbal epithelial cells in in vitro and animal studies,^20–25^ there are several drawbacks such as xenogenic infection (fibrin), repeat epithelial defects (hydrogels), limited availability (silk) and poor mechanical strength (collagen) that have prevented their widespread translation to the clinic. Therefore, hAM still remains the material of choice for human transplantation. One major advantage to the use of hAM is its anti-inflammatory property which greatly reduces the chance of rejection. Further, it induces neurotransmitters and growth factors that reduce vascularisation. Though PLGA does not exhibit these properties naturally, anti-inflammatory or anti-microbial agents can be easily incorporated into the material to improve its function. Amniotic membrane as a biological material provides a natural basement membrane with intact matrix components that allow the limbal epithelial cells to attach and grow retaining their stem cell properties. In an earlier study, we demonstrated that the PLGA membrane was also able to support the growth and differentiation of the limbal epithelial cells while retaining a subset of stem cell population.^15^ This property is key for the regeneration of the ocular surface as is evident in the present study.

The hAM carries the risk of disease transmission because its source is biological and variable. Also many surgeons do not have access to safe tissue banked sources of hAM. These limitations can be overcome with the use of synthetic polymers for preparing the PLGA membranes which can be produced in a repeatable manner under strict conditions. Unlike hAM, the degradation of which varies greatly due to its thickness, PLGA membranes degraded predictably within 8 weeks. We have demonstrated in our earlier studies that the PLGA degradation is neither toxic to the growing cells nor to the ocular surface following transplantation.^15 18^ It is well known that PLGA breaks down by hydrolysis and the break down products are well tolerated by the mucosal lining of the eye and do not induce any local or systemic adverse response. This was confirmed in a prior study in rabbits where we looked for any evidence of topical or systemic toxicity.^18^ This finding is consistent with the current report in humans.

There was however one finding, which requires further attention. Surgeons remarked that the membranes were much less flexible and more brittle than the hAM they were used to handling. The primary reason behind this is the extensive processing that was required to remove the residual solvents from the membrane to make it regulatory compliant for human use. This made the membranes more brittle which was not evident during the *in vitro* or animal testing since the membranes used in these studies did not have to be treated to the same extent as the ones used in the human study. Since the conclusion of the study, we have been examining this in detail to gain a better understanding of why these membranes were found to be relatively stiff at the point of use. This was not something that we had observed in our pre-clinical studies.

Accordingly, one of our current targets is to improve the flexibility of the PLGA membranes without affecting their ability to support explant outgrowth and corneal regeneration. To achieve this, we have been working on different approaches to control and tailor the mechanical properties of our membranes; these approaches include the use of different solvent systems during membrane manufacture as well as the use of biocompatible plasticising agents. This work is currently being prepared for publication.

## Conclusion

The PLGA membrane was well tolerated and provided sufficient support for the growth of the limbal epithelial cells to form a stable ocular surface. They degraded in a predictable manner by 8 weeks without causing any toxicity to the cells or the ocular surface.

## Data Availability

All data relevant to this study are included in the article.
