# Supplementary material for: Proof-of-concept study of electrospun PLGA membrane in the treatment of limbal stem cell deficiency
Source: BMJ Open Ophthalmol. 2021 Jul 26;6(1):e000762. doi: 10.1136/bmjophth-2021-000762 (PMC8314696; doi:10.1136/bmjophth-2021-000762)
Supplement: Supplementary data [file bmjophth-2021-000762supp001.pdf]

Supplementary figures

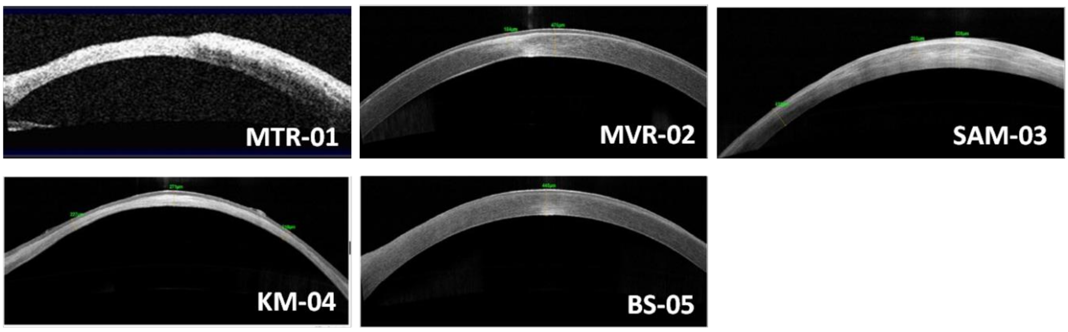

Supplementary figure 1: Shown in this figure are anterior segment OCT images of the patient's ocular surface at 6-12 months post-surgery.

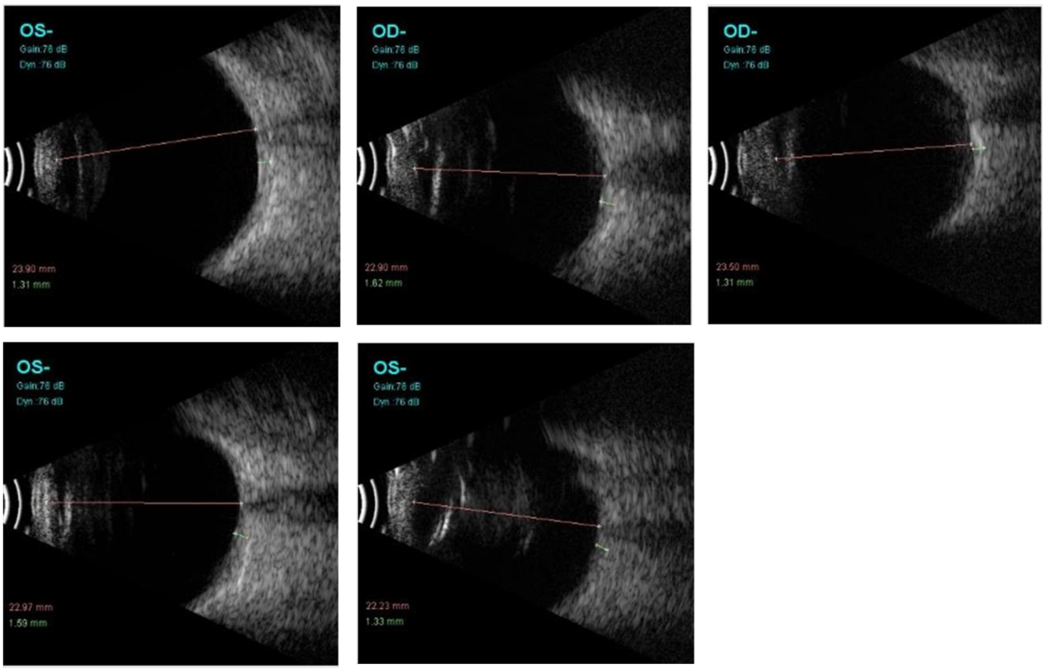

Supplementary figure 2: Shown here are the B-scan images of the patients treated eyes at 12 months follow up.

Supplementary table 1: Procedures performed on patients pre- and post- surgery

Activity	Screening	Day 0 (Day of Sx)	Day 1	Day 5 to 10	Day 21 to 30	Day 60 (± 15) days	Day 90 (± 15) days	Day 180/ (± 15) days	Day 360/ (± 15) days
Informed Consent	X								
Demographics	X								
Vitals	X							X	X
12-lead ECG (1)	X								
Medical History/Con meds	X								
Change in Medical history/Con Meds/baseline signs and symptoms		X	X	X	X	X	X	X	X
Detailed Ophthalmic Examination	X		X	X	X	X	X	X	X
Pain using VAS-Scale	X		X	X	X	X	X	X	X
Uncorrected and best corrected visual acuity by Snellen Chart	X				X	X	X	X	X
Slit-lamp biomicroscopy	X			X	X	X	X	X	X
Slit-lamp photography	X			X	X	X	X	X	X
IOP measurement digitally	X			X	X	X	X	X	X
IOP by Applanation Tonometry						X	X	X	X
Corneal Oedema (pachymetery)		X				X	X	X	X
Schirmer's test (5 minute) without anaesthesia	X					X	X	X	X
Record Baseline signs and symptoms	X								
Review Eligibility Criteria	X	X							
Review Discontinuation Criteria			X	X	X	X	X		
Assign UPIN		X							
Surgery		X							
Discharge from Hospital			X						
Safety Monitoring		X	X	X	X	X	X	X	X

**Clinical Laboratory Assessments: Haematology/Biochemistry/Urinalysis**	**X**						**X**	**X**	**X**
**Urine Pregnancy test (2)**	**X**								
**Record Final clinical assessment**									**X**

Supplementary table 2: Change in Best Correct Visual Acuity (BCVA) from Baseline to Final Visit

Patient ID	Pre-Sx		Post-Sx 30 days		Post-Sx 60 days		Post-Sx 90 days		Post-Sx 180 days		Post-Sx 360 days	
	OD	OS	OD	OS	OD	OS	OD	OS	OD	OS	OD	OS
MTR01	20/20	HM	20/20	HM	20/20	Defer	20/20	PL	-	-	20/20	PL
MVR02	CF/2 Mts	20/20	20/80	20/20	20/50	20/20	20/50	20/20	20/50	20/20	20/40	20/20
AM03	CF/1 Mts	20/20	CF/1 Mts	20/20	CF/2 Mts	20/20	Defer	20/20	Defer	20/20	20/400	20/20
KM04	20/20	HM	20/20	CF/ 20cm	20/20	CF/1 Mts	20/20	CF/1 Mts	20/20	CF/2 Mts	20/20	CF/2 Mts
BS05	20/20	20/400	20/25	20/100	20/20	20/100	20/20	20/100	20/20	20/60	20/20	20/80

HM: Hand movement; CF: Counting fingers; Sx: Surgery; PL: Perception of light; Defer: When acuity could not be measured due to tarsorraphy; -: patient had missed the follow up

Supplementary table 3: Change in Schirmer's test values from Baseline to Final Visit

Patient ID	Pre-Sx (mm)		Post-Sx 60 days (mm)		Post-Sx 90 days (mm)		Post-Sx 180 days (mm)		Post- Sx 360 days (mm)	
	OD	OS	OD	OS	OD	OS	OD	OS	OD	OS
MTR01	35	27	30	20	30	21	-	-	28	19
MVR02	18	26	28	26	20	18	12	20	15	5
AM03	20	27	23	26	*	35	*	*	24	20
KM04	26	18	29	28	33	33	33	35	30	24
BS05	27	20	*	*	20	18	13	14	10	14

-: patient missed this follow up; *: data was not collected during this visit; Sx: Surgery; Tear measurements were made for 5 minutes

Supplementary table 4: Change in Intraocular Pressure from Baseline to Final Visit

Patient ID	Pre-Sx		Post-Sx 30 days		Post-Sx 60 days		Post-Sx 90 days		Post-Sx 180 days		Post-Sx 360 days	
	OD	OS	OD	OS	OD	OS	OD	OS	OD	OS	OD	OS
MTR01	14	Dig. Higher	14	Dig. Normal	13	30	15	32	-	-	14	28
MVR02	Dig. Normal	13	14	18	19	15	15	12	14	12	14	12
AM03	Dig. Normal	15	18	14	Dig. Normal	13	Dig. normal	12	Dig. normal	12	15	14
KM04	20	15	14	18	18	19	16	Dig. Normal	16	18	12	18
BS05	17	19	12	Dig. Normal	14	Dig. Normal	12	Dig. Normal	13	14	10	10

Dig.: Digital measure; Sx: Surgery; -: missed follow up

Supplementary table 5: Hematology and Biochemistry Values

Hemoglobin (g/dL)				
Patient ID	Pre-Sx	90 days Post-Sx	180 days Post-Sx	360 days Post-Sx
MTR01	15.0	16.1	-	15.2
MVR02	13.8	14.4	ND	14
AM03	9.9	ND	9.8	10
KM04	14.6	14.9	15.3	15.0
BS05	14.8	13.7	14.8	13.3
Red blood cells (10^6/μl)				
MTR01	5.20	5.59	-	5.39
MVR02	4.25	4.47	4.59	4.22
AM03	3.86	4.06	4.17	3.73
KM04	4.94	4.8	5.0	4.9
BS05	5.10	4.36	4.52	4.21
White blood cells (x10^3/μl)				

**MTR01**	**10.1**	**9.4**	**-**	**8.4**
**MVR02**	**7.9**	**7.2**	**8.6**	**5.6**
**AM03**	**4.9**	**7.2**	**7.1**	**5.5**
**KM04**	**7.0**	**6.9**	**6.8**	**7.5**
**BS05**	**9.0**	**10.3**	**9.6**	**9.3**
**Platelet count (x10^3/µl)**				
**MTR01**	**186**	**192**	**-**	**202**
**MVR02**	**236**	**283**	**229**	**196**
**AM03**	**219**	**267**	**267**	**266**
**KM04**	**173**	**186**	**232**	**233**
**BS05**	**201**	**151**	**195**	**192**
**Neutrophils (10^3/µl)**				
**MTR01**	**4.71**	**5.58**	**-**	**5.74**
**MVR02**	**7.1**	**5.59**	**5.90**	**3.20**
**AM03**	**4.16**	**3.80**	**4.7**	**3.0**
**KM04**	**6.13**	**4.1**	**6.71**	**6.16**

**BS05**	**5.79**	**6.4**	**5.9**	**6.03**
**Lymphocytes (x10^3/μl)**				
**MTR01**	**4.50**	**3.60**	**-**	**3.10**
**MVR02**	**2.0**	**3.42**	**2.4**	**2.10**
**AM03**	**5.1**	**3.10**	**2.2**	**2.30**
**KM04**	**3.44**	**2.5**	**2.71**	**3.42**
**BS05**	**3.61**	**3.2**	**3.2**	**3.35**
**Monocytes (x10^3/μl)**				
**MTR01**	**0.70**	**0.40**	**-**	**0.40**
**MVR02**	**0.20**	**0.54**	**0.3**	**0.3**
**AM03**	**0.53**	**0.3**	**0.2**	**0.2**
**KM04**	**0.43**	**0.3**	**0.58**	**0.42**
**BS05**	**0.6**	**0.75**	**0.5**	**0.59**
**Hematocrit (%)**				
**MTR01**	**43.4**	**47.5**	**-**	**45.9**
**MVR02**	**40.3**	**43**	**41.9**	**40.0**

**AM03**	**41**	**32.3**	**36**	**34.8**
**KM04**	**41**	**40.8**	**46.3**	**42.8**
**BS05**	**39.6**	**38.7**	**41.2**	**ND**
**Serum Creatinine (mg/dL)**				
**MTR01**	**1.1**	**0.8**	**-**	**ND**
**MVR02**	**1.0**	**0.89**	**0.8**	**0.9**
**AM03**	**0.8**	**0.8**	**0.8**	**0.8**
**KM04**	**1.0**	**1.0**	**0.8**	**1.2**
**BS05**	**1.4**	**1.4**	**1.0**	**1.4**
**Blood urea /GLDH (mg/dL)**				
**MTR01**	**21**	**15**	**-**	**ND**
**MVR02**	**19**	**20**	**22**	**23**
**AM03**	**14**	**25**	**15**	**14**
**KM04**	**19**	**17**	**14**	**14**
**BS05**	**42**	**61**	**37**	**50**
**Random Blood Sugar (mg/dL)**				

MTR01	72	125	-	ND
MVR02	79	85	82	79
AM03	85	115	90	92
KM04	239	296	185	214
BS05	101	97	81	95
Sodium (mEq/L)				
MTR01	138	144	-	141
MVR02	ND	139	143	147
AM03	136	136	144	148
KM04	134	134	ND	140
BS05	138	138	145	151
Potassium (mmol/L)				
MTR01	4.2	4.1	-	4.2
MVR02	ND	4.2	3.9	3.8
AM03	4.0	3.5	3.7	3.5
KM04	3.7	3.9	ND	4.2

**BS05**	**4.3**	**4.2**	**3.8**	**4.6**
**Chloride (mEq/L)**				
**MTR01**	**100**	**101**	**-**	**100**
**MVR02**	**ND**	**102**	**106**	**104**
**AM03**	**106**	**99**	**106**	**103**
**KM04**	**103**	**104**	**ND**	**100**
**BS05**	**107**	**104**	**107**	**105**

ND: Not determined; -: Missed follow-up
